# Ocular Surgery

**Published:** 1885-08

**Authors:** 


### OCULAR SURGERY.

Dr. Landolt, of Paris, will commence this summer a course of practical lectures on operations on the eye. Should there be a sufficient number of American medical men who may wish to attend regularly, the Professor will have much pleasure in forming a separate class for them, at which the lectures will be delivered in English.

For further particulars, please address,

DR. LANDOLT, 4 Rue Volney,
Paris, France;

Or

DR. JOHN H. PAYNE, 415 Columbus Avenue,
Boston.

